# Synthesis of a multicomponent cellulose-based adsorbent for tetracycline removal from aquaculture water

**DOI:** 10.3762/bjnano.16.56

**Published:** 2025-05-27

**Authors:** Uyen Bao Tran, Ngoc Thanh Vo-Tran, Khai The Truong, Dat Anh Nguyen, Quang Nhat Tran, Huu-Quang Nguyen, Jaebeom Lee, Hai Son Truong-Lam

**Affiliations:** 1 Faculty of Chemistry, University of Science, Ho Chi Minh City 70000, Vietnam; 2 Vietnam National University, Ho Chi Minh City 70000, Vietnam; 3 Department of Chemistry, Chungnam National University, Daejeon 34134, Republic of Korea

**Keywords:** adsorption, aquaculture water, removal efficiency, response surface methodology, tetracycline antibiotic

## Abstract

Excessive use of tetracycline (TC) antibiotics in aquaculture, particularly in Vietnam, has contributed to environmental contamination and economic losses. To address this problem, we developed a novel cellulose-based multicomponent adsorbent material (PGC) synthesized from sodium carboxymethyl cellulose and investigated factors influencing its TC adsorption capacity. The synthesis process was optimized using parameters derived from the response surface methodology. The surface and structural properties of PGC were characterized, and the TC adsorption efficiency of PGC was assessed using high-performance liquid chromatography–mass spectroscopy (HPLC-MS). Elemental analysis of PGC identified four key mechanisms governing its endothermic TC adsorption mechanism: surface complexation, electrostatic interactions, hydrogen bonding, and CH–π interactions, with surface complexation between Ca^2+^ and TCs being dominant. Batch adsorption experiments conducted to examine the factors influencing adsorption capacity revealed that PGC achieved up to 70% TC removal efficiency at an adsorbent dosage of 40 mg and an initial TC concentration of 60 mg·L^–1^ at pH 6–7, reaching equilibrium after 12 h. The surface characteristics and structural properties of PGC were determined using various material characterization techniques, including FTIR, SEM, EDX, and BET. Verification experiments under optimal conditions confirmed that the adsorption process followed second-order kinetics and the Langmuir adsorption isotherm model. Under optimal experimental conditions, a maximum adsorption capacity (*q*_m_) of 123.2 mg·g^−1^ was estimated using the Langmuir isotherm model. These findings indicate that PGC demonstrates strong potential as an effective adsorbent for the removal of average 70% TC antibiotic residues, particularly oxytetracycline, chlortetracycline, TC, and doxycycline.

## Introduction

The aquaculture industry plays a crucial role in the global economy, particularly for coastal nations, including Vietnam. However, its multibillion dollar contributions are accompanied by the growing problem of excess antibiotic usage, notably tetracyclines (TCs), a widely used class of antibiotics in recent years [1–4]. Recent studies indicate that oxytetracycline (OTC), a TC derivative, is the predominant antibiotic used in Vietnam’s white leg shrimp farming industry, particularly during the 10–30 day and 30–45 day rearing periods [5]. The extensive use of OTC is primarily attributed to its broad-spectrum activity, rendering it effective in controlling various bacterial infections in shrimp. However, unregulated antibiotic usage poses significant risks, including the presence of antibiotic residues in seafood, which threaten human health. More broadly, overuse of antibiotics diminishes aquatic biodiversity and leads to substantial economic losses.

Nowadays, various methods, including adsorption, biological processing, photocatalysis, and electrochemical methods, have been used to remove antibiotics from contaminated water. However, these conventional treatment methods are restricted by costs, prolonged treatment durations, low adsorption efficiency, water matrices, and secondary pollutant formation, limiting their overall efficiency. A promising method for tackling tetracycline antibiotics involves membrane technologies such as osmosis membrane technology [6–7]. However, this approach presents significant upfront investment and recurring maintenance costs, while the contents of organic material and dissolved salts significantly affect the function of the membranes. Furthermore, challenges related to the draw solution and the necessity for integrating additional membrane processes for its regeneration remain key hurdles. Photochemical processes have also garnered significant research interest for tetracycline antibiotic removal, leveraging UV radiation [8–9]. While this approach offers cost-effectiveness, simplicity, and environmental benefits, its efficacy is strongly influenced by the compound’s adsorption spectrum, the sample matrix, and the radiation intensity. Activated carbon is a conventional approach [10–11], however, a major drawback of activated carbon is its incomplete recovery after adsorption. Because adsorption primarily relies on physical interactions such as hydrogen bonding interactions, electrostatic forces, and van der Waals forces, adsorbed antibiotics may desorb and reenter aquatic environments [12]. Moreover, activated carbon exhibits low selectivity and adsorption capacity. Among novel adsorbents, metal-organic frameworks [13] and molecularly imprinted polymers (MIPs) [14] are particularly notable for their high target specificity. Although MIPs are effective, their synthesis requires exceptional precision and is time-intensive. Meanwhile, magnetic solid-phase extraction columns [15] have been explored for TC removal; however, they are impractical for processing large sample volumes. These limitations have spurred the development of more effective and versatile adsorbents.

Modern adsorbents are available in diverse compositions. Moreover, they are easy to manufacture and generally both cost-effective and environmentally friendly. Cellulose-based adsorbents, in particular, have garnered increasing attention in recent years. For instance, Yao et al. used three-dimensional cellulose-based materials to remove various antibiotics from water, including TC, exhibiting high adsorption capacity and good reusability [16]. Moreover, three-dimensional cellulose-based aerogels, which feature high porosity and a large specific surface area, have demonstrated adsorption efficiency across a wide pH range [17].

Although previous studies have offered valuable insights, further research is needed to optimize the structural and compositional properties of materials to improve their performance. For instance, carboxymethyl cellulose (CMC), an anionic derivative of cellulose, is a linear polysaccharide consisting of anhydroglucose units linked by β-1,4-glycosidic bonds. The key distinction between CMC and cellulose is that some hydroxy groups in cellulose are replaced by carboxymethyl (–CH_2_COOH) groups. The introduction of carboxymethyl groups greatly enhances the water solubility of CMC relative to that of cellulose. CMC, recognized as one of the most promising cellulose derivatives, was first synthesized in 1918 [18]. Owing to its unique surface properties, high mechanical strength, abundance of raw materials, and cost-effective synthesis, CMC is now widely used in food, textile, pharmaceutical, and wastewater treatment industries.

This study aims to synthesize a cellulose-based multicomponent adsorbent material (PGC) using commercial sodium CMC, cross-linked with glutaraldehyde (GA) and polyvinyl alcohol (PVA); and cationized with Ca^2+^ and Zn^2+^ for the removal of TC from aquaculture effluents. Our approach involves optimizing the material’s synthesis using the response surface methodology, and a wide range of characterization methods was performed to assess the surface characteristics and morphology of the synthesized adsorbent. Additionally, the study examines the adsorption mechanism of TC on the material’s surface and evaluates the effects of pH value, adsorbent dosage, and matrix composition.

As a biodegradable and easily recoverable material derived from natural cellulose, this adsorbent offers a sustainable alternative to synthetic materials that pose environmental risks. In addition to wastewater treatment, this material could be utilized in medicine, pharmaceuticals, air purification, and environmental monitoring.

## Results and Discussion

### Experimental optimization

Conventional production and modification methods typically involve the manipulation of a single independent variable while holding all other variables constant [19]. However, chemical processes frequently involve a multitude of interacting factors, necessitating the simultaneous evaluation of potential interrelationships. To address this challenge, statistical experimental design methodologies, notably response surface methodology (RSM), have been developed. RSM, a robust integration of mathematical and statistical techniques, is extensively employed for process optimization and the elucidation of interactions among experimental variables, ultimately leading to enhanced results [20–21]. The application of RSM enables researchers to substantially decrease the number of experiments needed while simultaneously achieving a more thorough comprehension of the process under investigation and the identification of optimal operating parameters.

**Figure 1:**
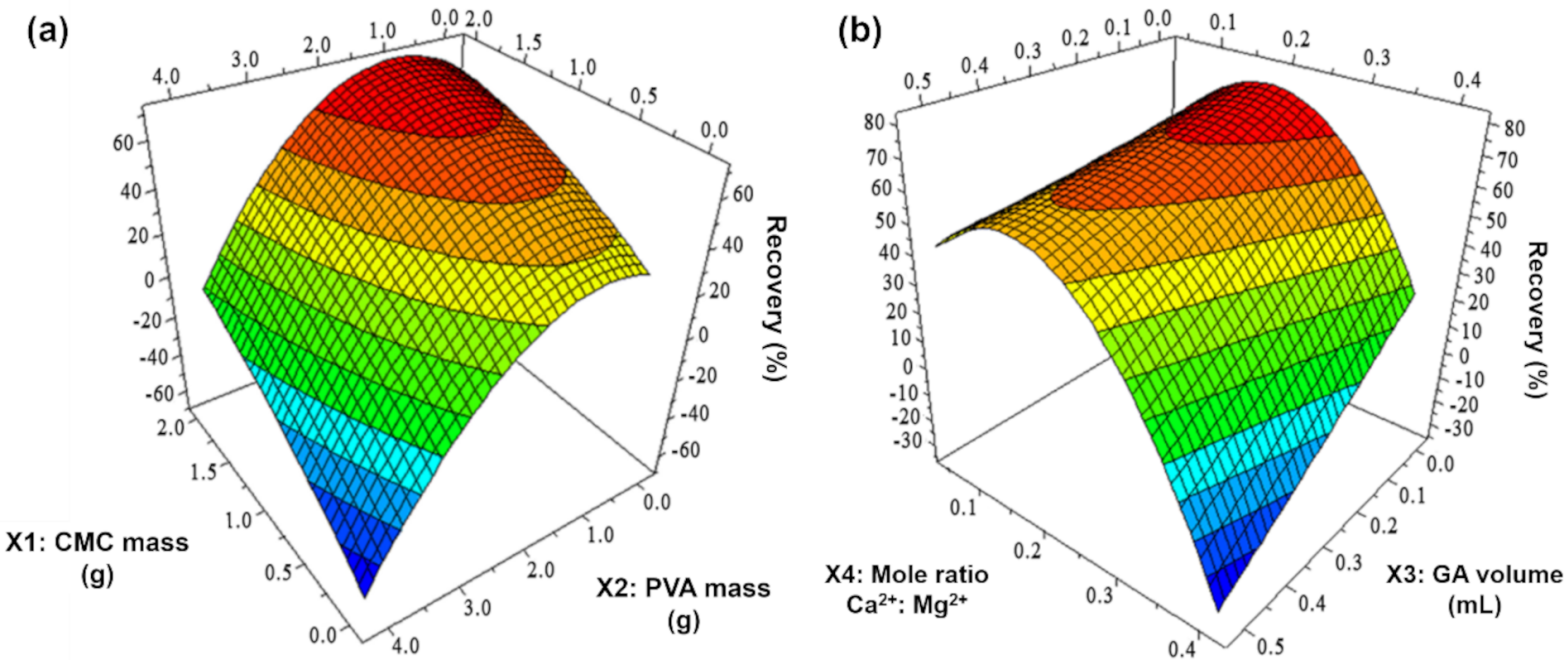
Response surface methodology (RSM) plots for synthesis optimization. (a) RSM plot showing the effect of X1 (CMC mass), X2 (PVA mass) and (b) RSM plot showing the effect of X3 (mole ratio Ca^2+^/Mg^2+^) and X3 (GA volume) on the optimum efficiency of the synthesis.

A response surface plot (Figure 1) was used to visualize variable interactions and determine optimal process parameters. As depicted in Figure 1a, the TC removal efficiency of the adsorbent decreases when both CMC mass (X1) and PVA mass (X2) increase simultaneously. This decline is expected, as increasing both CMC and PVA concentrations results in a highly viscous and non-homogeneous mixture, which deteriorates material quality and reduces adsorption capacity. When evaluating X2 independently, the optimal PVA concentration is determined to be below 2.0 g. When the PVA concentration exceeds this threshold, TC adsorption efficiency declines. This is because higher PVA levels hinder dissolution and mixing, particularly as viscosity increases. Similarly, TC adsorption efficiency also declines as GA concentration increases. At high concentrations, GA can dissolve PVA, compromising the material’s stability. This interaction significantly influences the model (p-value < 0.05), particularly through the GA volume (X3). The interaction of the mole ratio between Ca^2+^ and Mg^2+^ (X4) with other factors also has a significant effect on the model, yielding an optimal value of approximately 0.1. A substantial decrease in X4 leads to a corresponding decline in the dependent variable Y, particularly in the X1‒X4 and X3‒X4 interactions. This effect arises because a reduction in X4 decreases water solubility and hinders the formation of a homogeneous cellulose mixture. Additionally, lower Ca^2+^ concentrations impede chelate formation between Ca^2+^ and TC (Figure 1b). Response surface methodology (RSM) optimization in MODDE 5.0 identified the following optimal values for maximizing the objective function: X1 = 1.5 g, X2 = 1.0 g, X3 = 0.01 mL, and X4 = 0.1. These optimized parameters will be applied in the synthesis of an adsorbent for TC removal from water.

### Material characterization

#### FE-SEM and FTIR results

**Figure 2:**
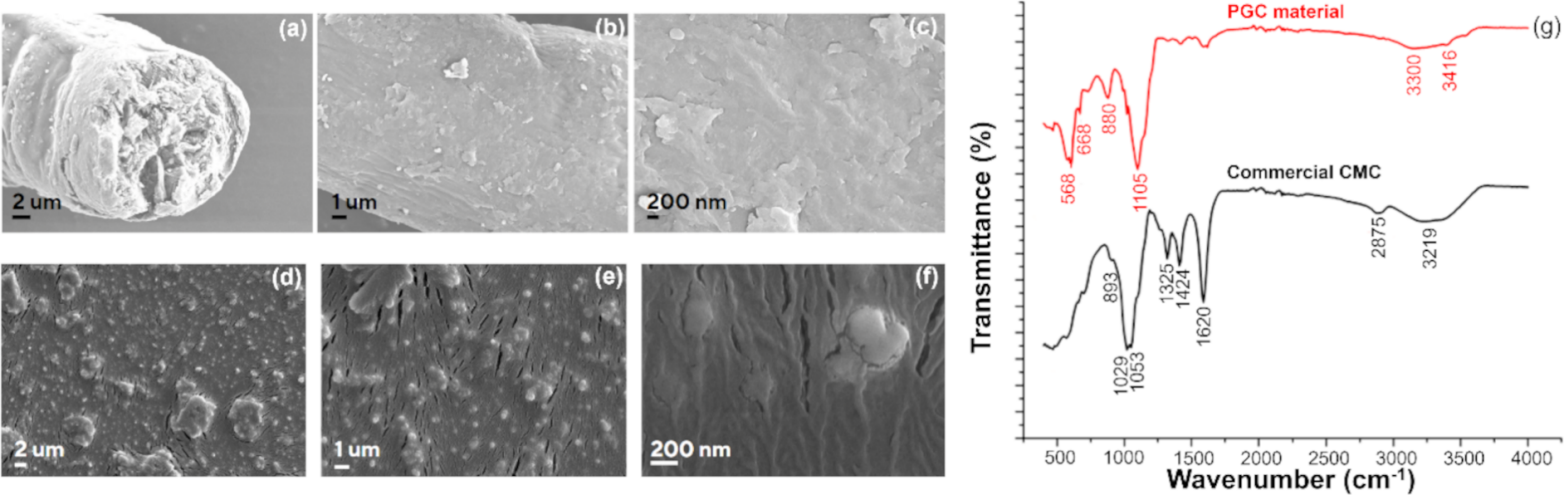
(a‒c) FE-SEM images of commercial CMC, (d‒f) FE-SEM images of PGC, and (g) FTIR spectra of commercial CMC and PGC.

Figure 2 presents comparative field-emission SEM (FE-SEM) images and FTIR spectra of commercial CMC and PGC. Notably, the FE-SEM analysis of PGC (Figure 2d–f) reveals significant morphological changes compared to pristine CMC (Figure 2a–c). Specifically, the PGC surface exhibits numerous, uniformly distributed spherical nanoparticles (≈200 nm in diameter), attributed to ZnO nanoparticles. The initial tubular structure of CMC is converted into a film-like structure owing to the lateral bonding effect of GA and PVA, as well as the dissolution of cellulose by Zn^2+^. The rough, wrinkled surface and cracks are likely due to the focused high-energy electron beam during the FE-SEM imaging process [22]. Larger agglomerates, possibly ZnSO_4_ residues, are also apparent, which aligns with the subsequent EDX results.

The FTIR spectrum (Figure 2g) of commercial CMC displays distinct adsorption bands at 3219, 2875, 1424, 1325, 1053, 1029 and 893 cm^−1^. The broad band from 3219 to 3406 cm^−1^ corresponds to O‒H stretching vibrations, reflecting the abundance of hydroxy and carboxyl groups in commercial CMC. The adsorption band at 2875 cm^−1^ represents symmetric stretching vibrations of the –CH_2_ group. The strong peak at 1620 cm^−1^ likely corresponds to asymmetric C=O stretching vibrations in carboxyl groups such as ‒COONa. The sharp, symmetric peaks at 1424 and 1325 cm^−1^ correspond to symmetric stretching vibrations of alkyl groups in CMC. The doublet at 1029 and 1053 cm^−1^ represents vibrations of pyranose rings formed during cellulose synthesis, as well as C‒O stretching vibrations. Meanwhile, the peak at 893 cm^−1^ corresponds to C‒O‒C stretching vibrations, which are characteristic of cellulose. These results are consistent with previous findings on commercial CMC [23–24].

Owing to lateral bonding, the characteristic peaks of CMC remain observable but exhibit shifts. For example, the alkyl group vibration peak shifts to 1424 cm^−1^, while the C‒O‒C stretching vibration peaks shift to 883 and 1105 cm^−1^. Meanwhile, the hydroxy group vibration peak becomes broader and less intense, shifting to the 3240‒3386 cm^−1^ region, suggesting the involvement of ‒OH groups in cross-linking. The intensity of the peak at 1325 cm^−1^ decreases significantly, while the peak at 1620 cm^−1^, corresponding to carbonyl (‒C=O) stretching in carboxyl groups, nearly disappears, indicating lateral bonding between PVA and GA. Additionally, the appearance of the peak at 668 cm^−1^ indicates the presence of a Zn‒O bond, while the peaks at 880 and 3416 cm^−1^ correspond to Zn‒OH vibrations, suggesting the involvement of Zn^2+^ in dissolving CMC. The sharp peak at 568 cm^−1^ corresponds to a Ca‒O bond [25].

#### EDX

**Figure 3:**
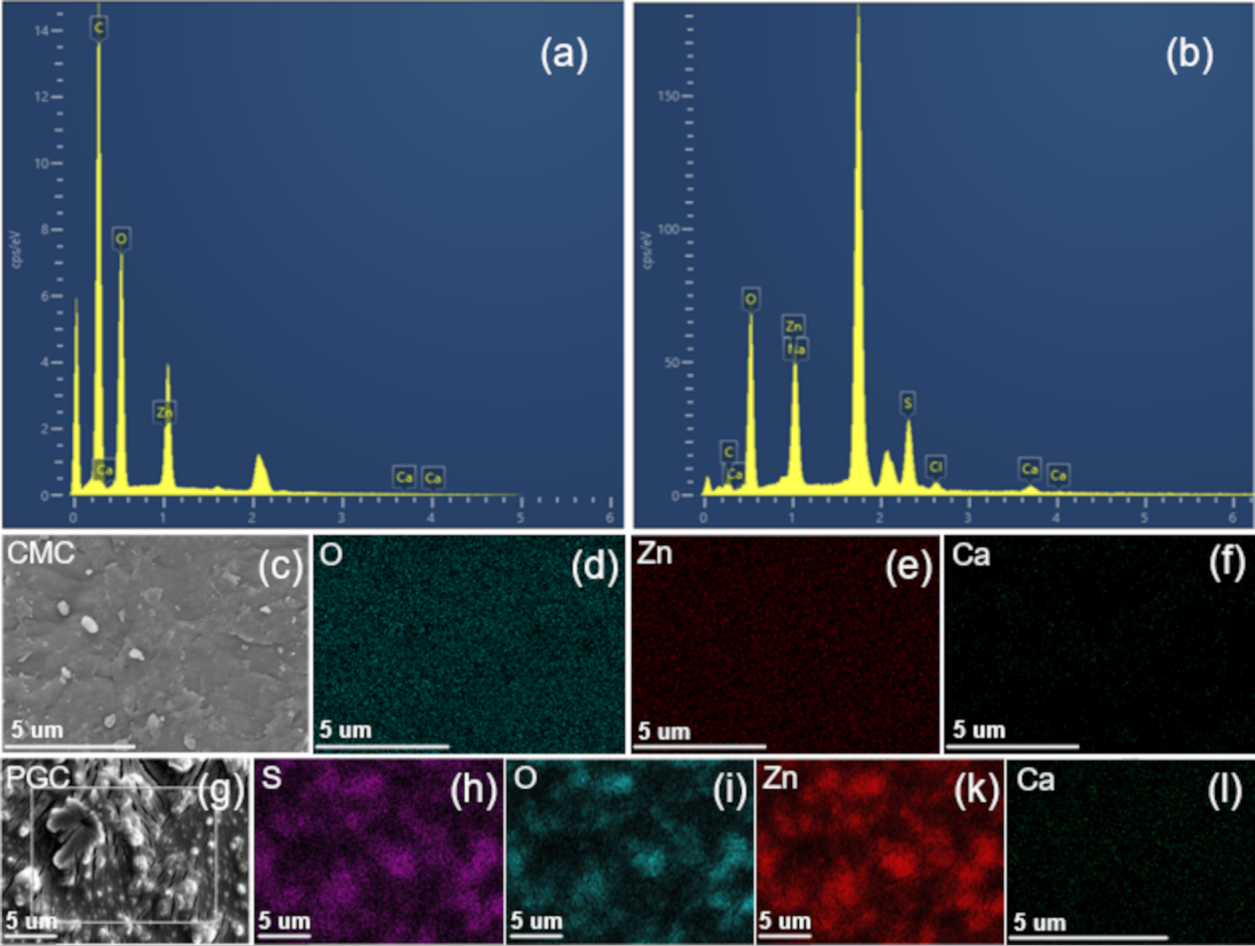
(a, b) EDX spectra and elemental compositions of commercial CMC and PGC, respectively; (c) morphology image of CMC; (d–f) elemental mapping images of commercial CMC; (g) morphology image of PGC; and (h–l) elemental mapping images of PGC.

EDX analysis revealed the elemental composition of the PGC material, as detailed in Figure 3a,b. Notably, the detection of elements, particularly Zn, in PGC confirms the role of Zn^2+^ ions in cellulose dissolution via hydrate bridge formation. Additionally, the presence of Zn enhances the TC adsorption capacity of PGC through a chemical adsorption mechanism.

According to our findings, Zn content increased significantly from 12.45% in pristine CMC to 22.24% in PGC, aligning with FTIR outcomes confirming the presence of Zn–O and Zn–OH bonds. Furthermore, the Ca content increased from 0.03% to 2.82%, accompanied by a rise in oxygen content. This increase suggests the involvement of GA and PVA in the cross-linking process, where the –OH groups in PVA and –CHO groups in GA contribute to the rise in oxygen content. Additionally, the detection of sulfur in PGC indicates the potential presence of residual ZnSO_4_ precursor. Figure 3c–l present significant changes in the elemental distribution of O, S, Zn, and Ca in PGC compared to pristine CMC. These elements display a higher density on the surface of PGC.

#### Brunauer–Emmett–Teller method

Surface area characterizations of PGC determinded by the Brunauer–Emmett–Teller (BET) method are shown in Table 1. Specifically, the specific surface area of PGC is 1.3543 m^2^·g^−1^; the pore volume is 0.000641 cm^3^·g^−1^ and the average pore diameter is 18.93 Å.

**Table 1:** Surface area characterizations of PGC determinded by BET method.

Surface area (m^2^/g)	

Single point surface area at *P*/*P*_0_ = 0.249674088	1.2965
BET surface area	1.3543
t-Plot micropore area	0.2295
t-Plot external surface area	1.1248

Pore volume (cm^3^/g)	

Single point adsorption total pore volume of pores less than 34.994 Å diameter at *P*/*P*_0_ = 0.398928826	0.000641
t-Plot micropore volume	0.000101
BJH adsorption cumulative volume of pores between 17.000 Å and 3,000.000 Å diameter	0.000461

Pore size (Å)	

Adsorption average pore diameter	18.930
BJH adsorption average pore diameter	20.758

### Investigation of factors influencing the maximum TC adsorption capacity of the synthetic material

#### Effect of initial pH

**Figure 4:**
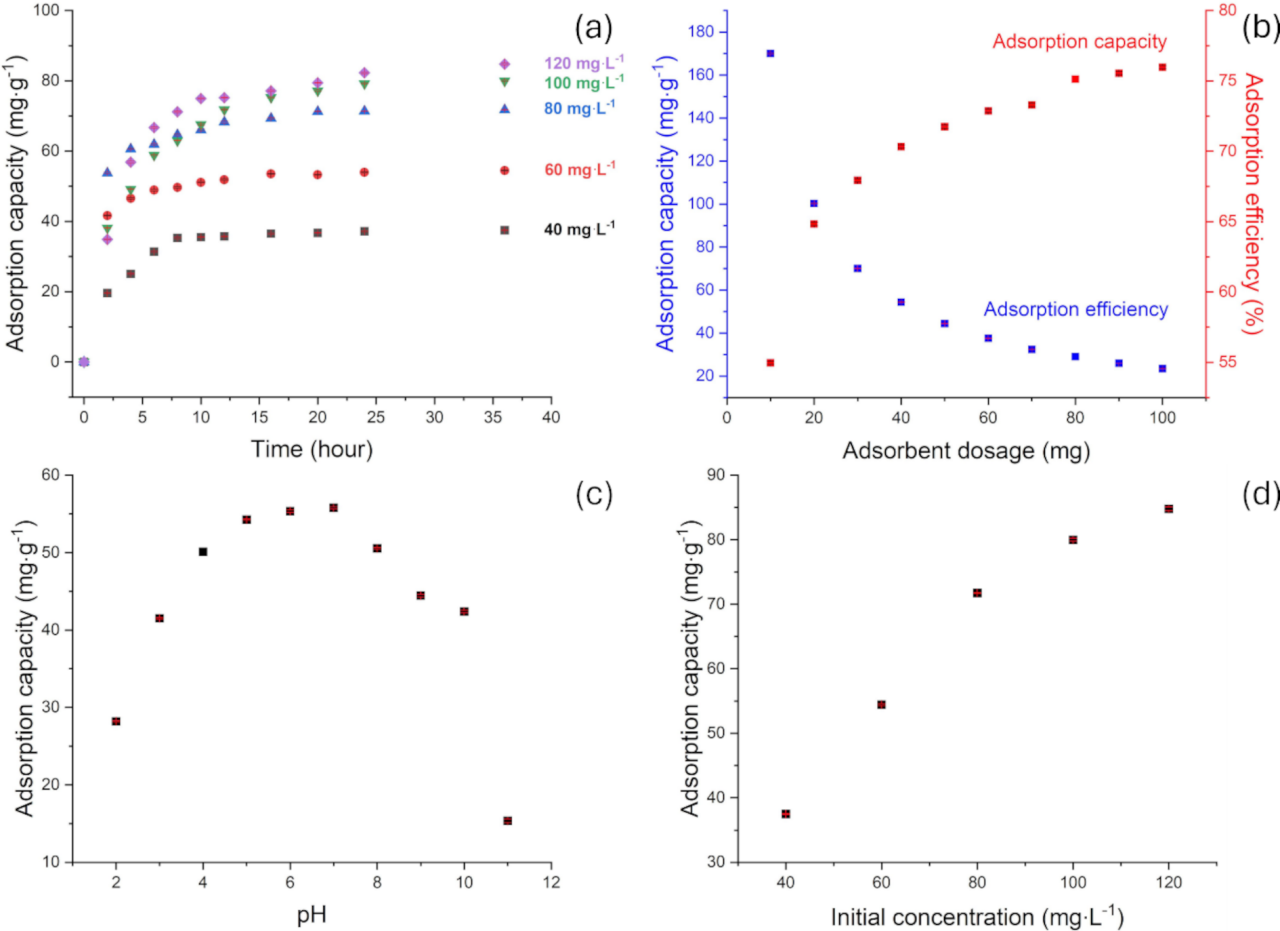
(a) Effect of adsorption time and initial concentration on the adsorption capacity of PGC. (b) Effect of adsorbent dosage on the adsorption capacity and adsorption efficiency of PGC. (c) Effect of pH on the adsorption capacity of PGC. (d) Effect of initial concentration on the adsorption capacity of PGC.

The effect of pH on the TC adsorption capacity of PGC is illustrated in Figure 4c. Specifically, the adsorption capacity increases significantly between pH 3 and 7, peaking at pH 7. Beyond this point, it decreases rapidly, with the sharpest decline observed between pH 10 and 11.

This occurs because, as pH increases, particularly around pH 6.8, keto-enol groups serve as preferential sites for chelate formation between TC and Ca^2+^ in a 1:3 ratio of Ca^2+^ to TC [26]. In this pH range, reduced competition between H^+^ ions and TC for adsorption sites, along with the ionization of hydroxy groups and the subsequent formation of hydrogen bonds with TC molecules, results in a rapid increase in adsorption capacity. Beyond pH 7, adsorption capacity decreases sharply as TC transforms into negatively charged anions, causing repulsive interactions with oxygen-containing functional groups on the PGC surface. Further, at pH 7.5 and above, the chelate complex between Ca^2+^ and TC preferentially forms at a 1:1 ratio [27]. Hence, the pH range between 6 and 7 is selected as optimal for TC adsorption and will be used in subsequent investigations.

#### Effect of initial TC concentration and time

As depicted in Figure 4a, the adsorption capacity (*q*_e_) increases with higher initial concentrations of TC. Specifically, at low initial concentrations, adsorption capacity is low owing to the incomplete diffusion of TC molecules into the material structure. However, at higher initial concentrations, a larger concentration gradient drives TC diffusion into the PGC surface, resulting in a rapid increase in adsorption capacity. Notably, most of the adsorption occurs within the first 12 h, during which 89–95% of TC is adsorbed. In the first 8 h, the adsorption rate of TC increases rapidly at all concentrations but decreases significantly afterward. This occurs because, during the initial stage, numerous vacant adsorption sites on the surface allow for the easy adsorption of TC. As TC molecules fill the vacant adsorption sites, the adsorption rate decreases over time until equilibrium is reached after 12–16 h.

As depicted in Figure 4a, the adsorption efficiency at 60 mg·L^−1^ TC increases more rapidly in the first 12 h than at other concentrations. Therefore, a concentration of 60 mg·L^−1^ was selected for further investigations.

#### Effect of adsorbent dosage

An adsorption experiment was performed using 10 different adsorbent dosages at an initial TC concentration of 60 mg·L^−1^ and pH 6–7. Figure 4b presents the effect of adsorbent dosage on the TC adsorption capacity and efficiency of PGC. Notably, as the adsorbent dosage increases, TC adsorption capacity decreases, whereas adsorption efficiency improves owing to the greater surface area available for TC adsorption. Adsorption efficiency increases rapidly as the adsorbent dosage rises from 10 to 40 mg, but beyond this point, the rate of increase becomes negligible. Doubling the adsorbent dosage to 80 mg results in an increase of no more than 10% in both TC adsorption capacity and removal efficiency. However, the amount of TC adsorbed per unit mass of adsorbent decreases as dosage increases. Therefore, an adsorbent dosage of 40 mg is selected for subsequent studies.

#### Adsorption isotherms

**Figure 5:**
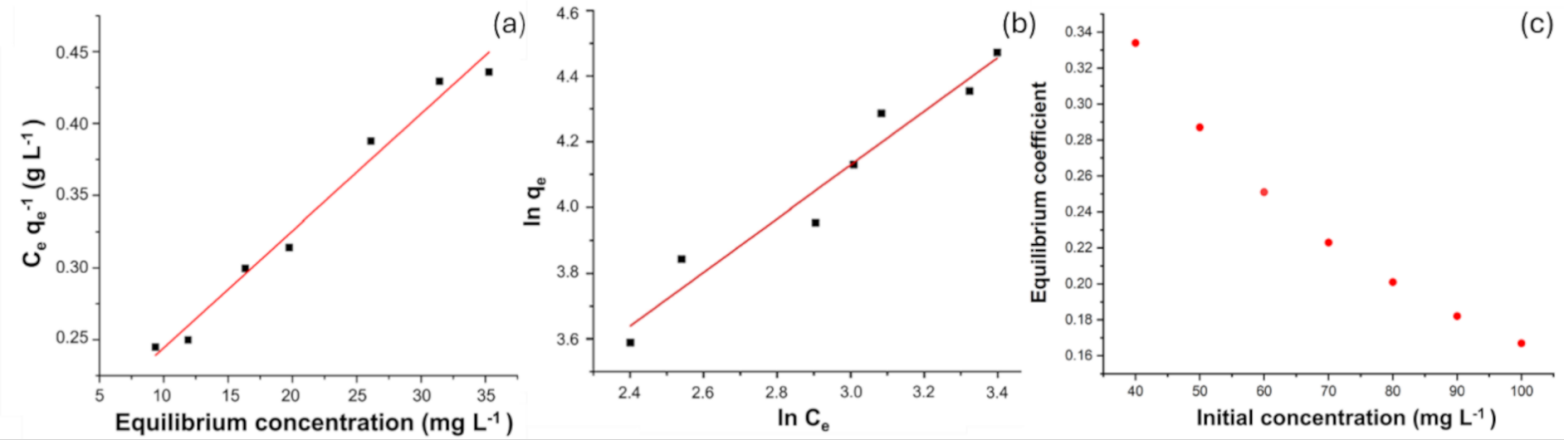
(a) Langmuir adsorption isotherms. (b) Freundlich adsorption isotherms. (c) Variation of the equilibrium constant *R*_L_ as a function of initial concentration.

An adsorption test was conducted at pH 6–7 using a 40 mg adsorbent dosage with varying initial TC concentrations. Linear regression analysis was applied to *C*_e_*/q*_e_ and *C*_e_ for the Langmuir model (Figure 5a) and to ln *q*_e_ and ln *C*_e_ for the Freundlich model (Figure 5b). To assess whether TC adsorption onto PGC follows the monolayer adsorption mechanism described by the Langmuir model, the degree of fit was evaluated using the equilibrium coefficient *R*_L_. Notably, the *R*_L_ values, calculated in Table 2 and presented in Figure 5c, range from 0.167 to 0.334, indicating that TC adsorption onto PGC is favorable and conforms to the Langmuir isotherm model.

**Table 2:** Characteristic parameters and *R*^2^ coefficients of the Langmuir and Freundlich models according to the linearized models.

	Langmuir model			Freundlich model			
Adsorbent	*R* ^2^	*q*_m_ (mg·g^−1^)	*K*_L_ (L·mg^−1^)	*R*_L_	*R* ^2^	*K*_F_ (L·g^−1^)	*n*

PGC	0.9823	123.2	0.05	0.167–0.334	0.9495	5.36	1.23

The higher *R*^2^ value for the Langmuir model compared to that for the Freundlich model (Table 2, Figure 5a,b) suggests that the Langmuir model better describes TC adsorption onto PGC. This finding indicates that TC adsorption onto PGC occurs as monolayer adsorption on a homogeneous surface.

#### Adsorption kinetics

Experimental data derived from the analysis of the effect of contact time and initial TC concentration on adsorption capacity were used to study the kinetics of TC adsorption using first-order and second-order kinetic models. Table 3 and Table 4 present the first-order and second-order kinetic equations, respectively. Although the first-order kinetic model yields relatively high *R*^2^ values (0.89–0.98), the equilibrium adsorption capacity calculated based on the model equations deviates significantly from experimental values. Therefore, the first-order kinetic model is unsuitable for describing TC adsorption onto PGC.

**Table 3:** First-order kinetics equations and *R*^2^ values.

Concentration (mg·L^−1^)	First-order kinetics equations	*R* ^2^

40	*y* = −0.1850*x* + 3.0307	0.9206
60	*y* = −0.1651*x* + 3.0311	0.8987
80	*y* = −0.1951*x* + 3.6012	0.9514
100	*y* = −0.1746*x* + 4.2163	0.9840
120	*y* = −0.1285*x* + 3.9464	0.9242

**Table 4:** Second-order kinetics equations and *R*^2^ values.

Concentration (mg·L^−1^)	Second-order kinetics equations	*R* ^2^

40	*y* = 0.0275*x* + 0.0303	0.9971
60	*y* = 0.0181*x* + 0.0113	0.9995
80	*y* = 0.0137*x* + 0.0103	0.9992
100	*y* = 0.0118*x* + 0.0245	0.9946
120	*y* = 0.0113*x* + 0.0212	0.9952

In contrast, the pseudo-second-order kinetic model exhibits high *R*^2^ values (>0.99) and excellent agreement between calculated and experimental equilibrium adsorption capacities, indicating that it better describes TC adsorption onto PGC. This suggests that the adsorption process is predominantly chemisorption.

#### Mechanism

**Figure 6:**
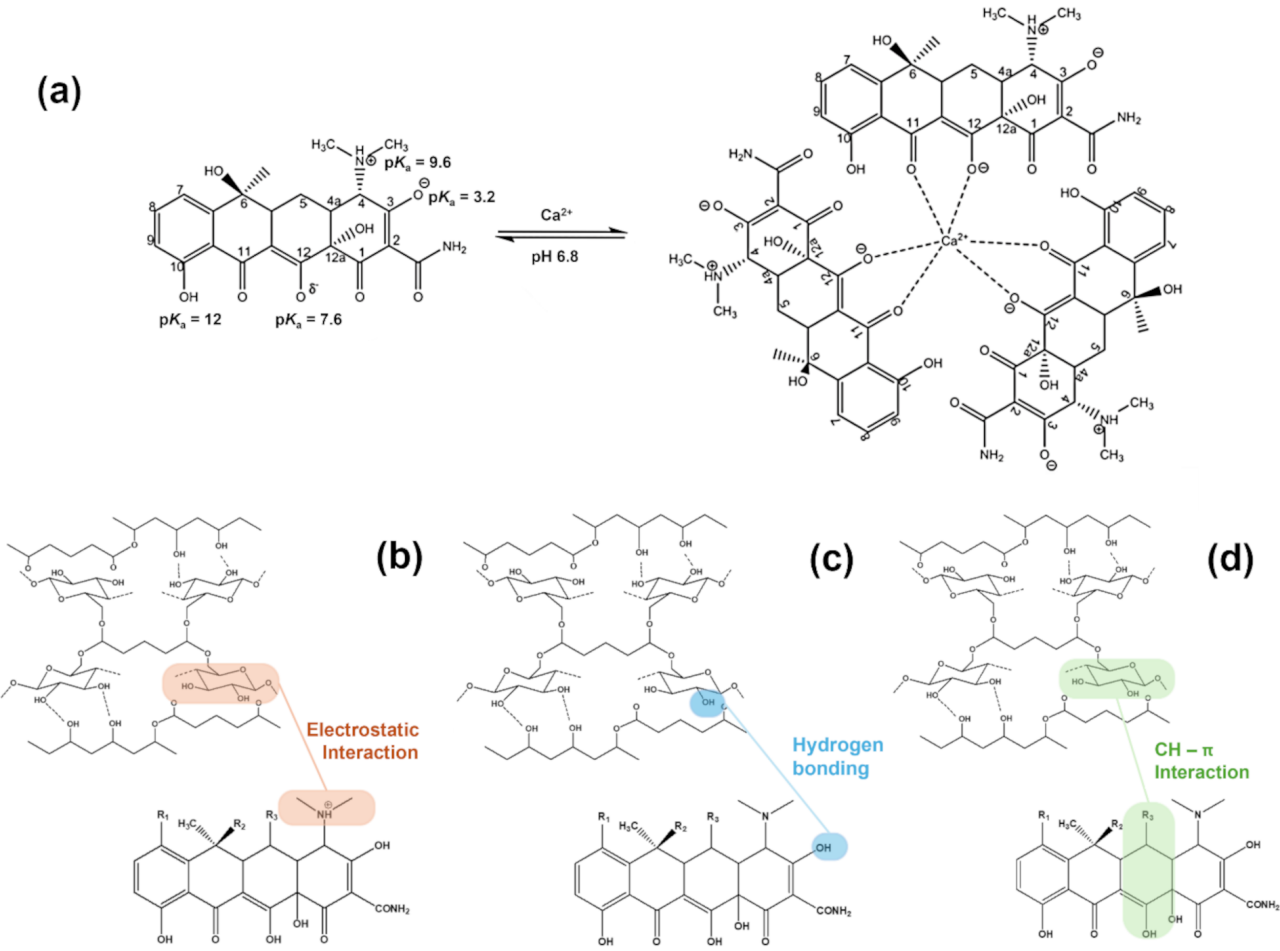
(a) Structure of the Ca^2+^‒TC complex formed at pH 6.8. Adsorption mechanism of TC onto the PGC surface through (b) electrostatic interaction, (c) hydrogen bonding, and (d) CH–π interaction.

Elemental analysis of the synthesized PGC material confirmed the presence of Ca^2+^, indicating the potential formation of Ca^2+^–TC complexes. Notably, the formation of these complexes depends on pH, as specific sites on the TC structure undergo protonation before ionic bonding with Ca^2+^ in the PGC matrix. Among the four ionizable functional groups in TC, the primary site remains predominantly protonated (86%) at pH 6.8 [26,28–29]. However, Ca^2+^ coordination at this site is expected to shift the ionization equilibrium toward the β-keto enolate form, leading to the deprotonation of TC molecules and complex formation with Ca^2+^. Experimental results indicate that a 1:3 Ca^2+^/TC complex is favored at pH 6.8 (Figure 6a) [29].

The adsorption mechanism of TC onto PGC is illustrated in Figure 6b,c,d. This mechanism involves van der Waals forces, which primarily represent electrostatic interactions. Notably, TC is an aromatic organic compound with an amino group that can accept protons (H^+^) from the environment, acquiring a positive charge. The CMC network within PGC includes oxygen-containing functional groups (‒OH, ‒C=O, and ‒COOH), which confer a negative charge, enabling electrostatic interactions (Figure 6b). FTIR spectroscopy (Figure 2g) confirms the presence of these oxygen-containing functional groups on PGC, supporting this explanation. Thus, electrostatic attraction between the positively charged TC–N^+^ complex and the negatively charged PGC material drives the adsorption process. Additionally, hydroxy groups may facilitate hydrogen bond formation between PGC and TC (Figure 6c). Furthermore, the aromatic ring of TC contains conjugated double bonds, while the hexagonal network of PGC contains ‒CH groups, enabling CH–π dispersion interactions [30] (Figure 6d).

#### Removal of antibiotics from water sample via PGC material

As shown in Table 5, approximately 70% and 60% of antibiotics content (including tetracyline (TC), oxytetracycline (OTC), chlortetracycline (CTC), and doxycycline (DOX)) in standard solution and sample solution, respectively, was successfully eliminated from water during a single-stage PGC treatment.

**Table 5:** Antibiotics treatment performance in water sample after one stage of the PGC process.

Matrix	Removal efficiency (%)	

standard solution	OTC	71.03 ± 0.96
	TC	72.24 ± 1.21
	CTC	82.68 ± 0.85
	DOX	86.98 ± 0.36

sample solution	OTC	46.07 ± 2.31
	TC	66.39 ± 0.52
	CTC	65.84 ± 1.53
	DOX	86.39 ± 0.67

PGC material ultilization promotes the valorization of agricultural waste, offering an effective strategy for its reuse, particularly relevant for agricultural countries like Vietnam. This provides an abundant and low-cost source of raw materials and mitigates environmental issues linked to the burning or improper handling of agricultural by-products. Notably, PGC also shows strong potential for commercialization as an efficient water treatment solution. Nonetheless, the presence of humic and fulvic acids in the sample matrix may interfere with the adsorption process, presenting a significant obstacle to the effective use of PGC for TC removal.

## Conclusion

This study developed and implemented a synthesis strategy for a cellulose-derived adsorbent material (PGC) to remove TC antibiotics (tetracyline, oxytetracycline, chlortetracycline, and doxycycline) from aquaculture water. RSM was used to determine the optimal synthesis parameters for PGC, which were CMC mass (1.5 g), PVA mass (1.0 g), GA volume (0.01 mL), and Ca^2+^/Zn^2+^ molar ratio (0.1). FTIR, EDX, FE-SEM, and BET analyses were used to assess the cross-linking performance of GA and PVA and to elucidate the role of Zn^2+^ in cellulose dissolution. The adsorption of TC onto PGC was explained by the formation of Ca^2+^–TC chelate complexes, as well as electrostatic interactions, CH–π interactions, and hydrogen bonding interactions between the material surface and TC. Additionally, the effects of contact time, pH, initial concentration, and adsorbent dosage on the TC adsorption capacity of PGC were investigated. The results indicated that equilibrium was reached after 12 h, with an optimal pH of 6–7, an adsorbent dosage of 40 mg, and an initial concentration of 60 mg·L^−1^ TC. TC adsorption onto PGC followed pseudo-second-order kinetics and conformed to the Langmuir isotherm model. Additionally, preliminary tests in real water samples revealed that fulvic acid and humic acid in the water matrix affected the adsorption process. Owing to its high efficiency, eco-friendliness, versatility, and up to 70% removal efficiency of tetracyline, oxytetracycline, chlortetracycline, and doxycycline, the synthesized PGC material shows great potential for addressing environmental challenges and promoting sustainable development. The PGC adsorbent, with higher porosity, enhanced selectivity, more hydrophobicity, and a simple synthesis process, along with cost-efficiency and high adsorption capacity, holds promise as an effective adsorbent for the treatment of aquaculture wastewater. Overall, this study lays the groundwork for future research on synthesizing adsorbents from sustainable, cellulose-based materials derived from agricultural waste.

## Experimental

### Materials

Tetracycline hydrochloride (97.2%), oxytetracycline dihydrate (98%), and chlortetracycline hydrochloride (94.6%), all sourced from the Institute of Drug Quality Control, Ho Chi Minh City (Vietnam), were used as reference antibiotics in this study. Additional reagents included ciprofloxacin and enrofloxacin (both from Pharmaceutical Joint Stock Company of February 3rd, Vietnam), methanol (Merck, Germany), sodium carboxymethyl cellulose (Zhanyun, China), PVA (95.5–96.5% hydrolyzed, *M*_W_ ≈ 85,000–124,000, Thermo Scientific Chemicals, USA), GA (50%) (Zhanyun, China), calcium chloride anhydrous (Xilong, China), and zinc sulfate heptahydrate (Xilong, China). All reagents were of analytical grade, and deionized water was used for all experiments.

### Experimental optimization

MODDE 5.0 software was employed to identify key influencing factors and optimize the synthesis process using RSM. The independent variables included CMC mass (X1) [31], PVA mass (X2), and GA volume (X3) [32], along with the molar ratio of Ca^2+^ and Zn^2+^ (X4) [25].

Polyvinyl alcohol (PVA), characterized by a high density of hydroxy groups attached to its polymer chain, is widely used as a binding agent in material synthesis. PVA promotes chemical cross-linking between CMC molecules by interacting with acidic and/or basic functional groups under thermal conditions [33–34]. This cross-linking occurs when the polymer’s free hydroxy groups interact with the functional groups of the cross-linking agent, reducing the polymer’s water solubility while increasing its stiffness and chemical stability [35–36].

Glutaraldehyde (GA), a linear five-carbon dialdehyde, is regarded as a more effective cross-linking agent compared to monoaldehydes (e.g., formaldehyde) and other dialdehydes (C_2_ to C_6_) [37]. GA and PVA have been used as cross-linking agents in CMC-based materials to enhance selectivity, stability, and mechanical properties [38]. This method is both cost-effective and highly efficient in strengthening materials while improving their mechanical strength and hydrophobicity.

Recent studies have revealed that inorganic salt mixtures, such as zinc chloride and calcium chloride, effectively dissolve cellulose, facilitating the fabrication of cellulose membranes for gas separation and organic pollutant removal [38–39]. Specifically, in a cellulose solution, Ca^2+^ cross-linking with Zn‒cellulose chains enhance the mechanical properties of the resulting membranes. These ions can be incorporated into the cellulose polymer matrix with an appropriate ratio, forming a controlled hydrogen bonding network that strengthens connectivity in the overall polymer network [40].

### Preparation of PGC

**Figure 7:**
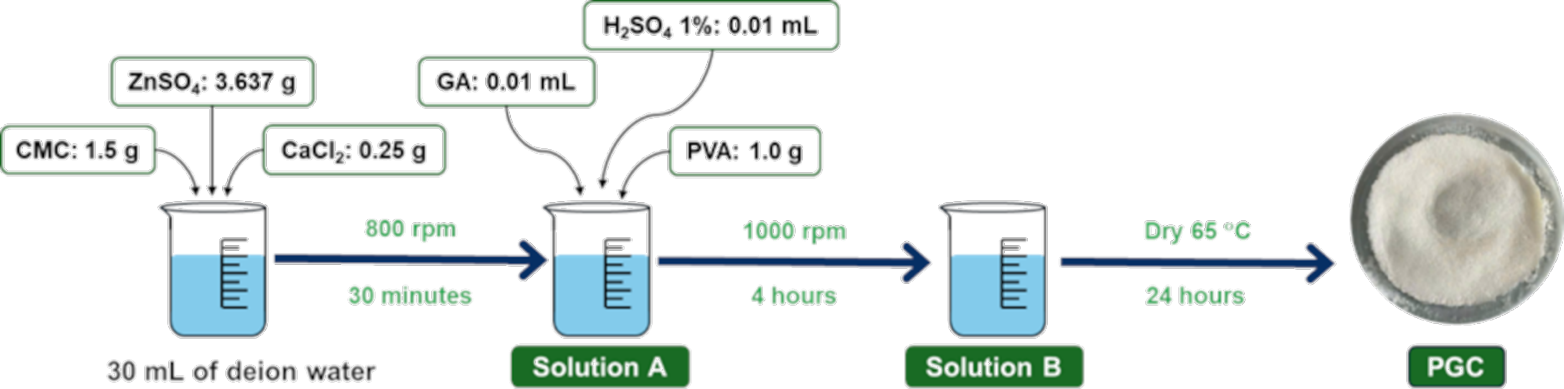
Synthesis procedure for PGC.

As illustrated in Figure 7, CMC was selected as the base material for the synthesis of PGC, in combination with the presence of the following agents: PVA, which acts as a binder by facilitating chemical cross-linking of CMC molecules through interactions with functional acidic and basic groups, the cross-linking agent glutaraldehyde (GA), and an inorganic ion mixture (Ca^2+^ and Zn^2+^), which enhances the mechanical properties of the material by promoting the formation of a controlled hydrogen bonding network, thereby reinforcing the polymer matrix. To prepare PGC, 1.5 g of CMC, 0.25 g of CaCl_2_, and 3.637 g of ZnSO_4_ were added to a beaker containing 30 mL of distilled water, where they were completely dissolved under magnetic stirring at 800 rpm for 30 min, forming solution A. Subsequently, 1.0 g of PVA and 0.1 mL of 1% H_2_SO_4_ were added, and the solution was stirred until a homogeneous mixture was obtained. Next, 0.01 mL of GA was added, and the stirring speed was increased to 1,000 rpm, continuing for 4 h. Finally, the solution was dried at 65 °C for 24 h, yielding the PGC adsorbent material for further use.

### Characterization of PGC

#### FE-SEM and EDX

Field-emission SEM analysis was performed using a Merlin Compact instrument (Carl Zeiss, Jena, Germany) with an SE2 detector. The sample was mounted on a clean silicon wafer and coated with a nanoscale platinum layer using an ion sputter coater (Q150T Plus, Quorum Technologies, UK). EDX analysis was conducted using an Aztec Energy X-MaxN system (Oxford Instruments, UK) at an acceleration voltage of 5 kV and a working distance of 8.5 mm.

#### FTIR

FTIR analysis was performed using a Spectrum Two FTIR spectrometer (PerkinElmer, MA, USA) equipped with a LiTaO_3_ detector and an attenuated total reflectance sampling accessory. The scanning range was 400–4000 cm^−1^, with a total acquisition time of 60 s.

#### Brunauer–Emmett–Teller method

A 0.27 g PGC sample was analyzed at the Institute of Chemical Technology, Ho Chi Minh City, over 3 h. The specific surface area of this sample was determined using N_2_ adsorption‒desorption isotherms at 77.3 K under controlled pressure conditions. Before analysis, the sample was degassed at 150 °C for 2 h and 30 min under an N_2_ atmosphere.

#### High-performance liquid chromatography-mass spectroscopy (HPLC–MS/MS)

The HPLC-MS/MS system consisted of an AB Sciex 4000 QTRAP mass spectrometer equipped with a Turbo Ion Spray source, which was operated in both positive mode and negative mode (QTRAP®4000, AB SCIEX, Framingham, MA, USA). The analyses of the tetracyclines were performed using a Sunfire C18 column (150 × 2.1 mm i.d., 5.0 mm particle size) from Waters (Milford, MA, USA), and the mobile phase consisted of ACN and 0.1% FA, delivered at 0.25 mL·min^−1^.

#### Factors influencing adsorption capacity

Experiments were conducted to evaluate factors affecting the adsorption capacity of PGC and determine optimal conditions. The investigated factors included initial pH, initial concentration and time, adsorbent dosage, adsorption isotherms, and adsorption kinetics.

## Data Availability

The data collected during the current study will be available from the corresponding author on reasonable request.
